# Leveraging Classical Virology and High Throughput Sequencing for Viral Discovery Using a Historical Viral Collection

**DOI:** 10.3390/v17111513

**Published:** 2025-11-18

**Authors:** Mark Sistrom, Matthew Neave, Ancy Joseph, Kim Newberry, Hannah Andrews, Cathy Shilton, Vidya Bhardwaj, Richard Weir

**Affiliations:** 1 Northern Territory Government Department of Health, Territory Pathology, Royal Darwin Hospital, Tiwi, NT 0810, Australia; 2 Northern Territory Government Department of Industry, Trade and Tourism, Berrimah Veterinary Laboratories, Darwin, NT 0801, Australia; hannah.andrews@nt.gov.au (H.A.); cathy.shilton@nt.gov.au (C.S.); vidya.bhardwaj@nt.gov.au (V.B.); richard.weir@nt.gov.au (R.W.); 3 Research Institute for the Environment and Livelihoods, Charles Darwin University, Darwin, NT 0810, Australia; 4 Australian Centre for Disease Preparedness, Commonwealth Scientific and Industrial Research Organisation (CSIRO), Geelong, VIC 3220, Australia; matthew.neave@csiro.au (M.N.); ancy.joseph@csiro.au (A.J.); kim.newberry@csiro.au (K.N.)

**Keywords:** zoonotic, *Hapavirus*, *Orbivirus*, *Orthobunyavirus*, cattle, arbovirus, Australia

## Abstract

Northern Australia has long been a hotbed of arboviral discovery, and collections of viral isolates from Northern Australia represent an invaluable resource for both our knowledge of viral diversity and for disease preparedness and treatment. While discovery of novel viruses via classical virology methods is on the decline, next generation sequencing offers the possibility to speed up viral discovery, albeit at the expense of the collection of valuable life history data. By sequencing unknown isolates from historical viral collections, we may leverage both the rich data collected through classical virology and the power of identification using contemporary sequencing technologies. In the present study, we sequenced 76 historical viral isolates held at the Berrimah Veterinary Laboratory in Darwin, northern Australia, for which serological typing had yielded ambiguous results. We determined that 43 of these isolates belong to the genera *Hapavirus, Orbivirus*, and *Orthobunyavirus*. Several of these isolates are putatively novel genotypes or potential taxa, which has significant potential implications for human and animal health. This study demonstrates the utility of historical collections for viral discovery and characterisation and how considerable past efforts to isolate and characterise viruses can be enhanced using next generation sequencing approaches.

## 1. Introduction

Arbovirus discovery has a rich and storied history [1] and has played a critical role in the documentation and development of knowledge of viral diversity broadly speaking. While there has been a decline in the discovery of novel viruses via classical methods of virology [1], the development of high throughput sequencing has facilitated a suite of new methods for the identification and characterisation of novel viruses. While the pace of viral discovery offered by next generation sequencing techniques is unequivocally more rapid than classical techniques [2] and often allows for the ascertainment of genomic data for novel viruses, it often negates the ability to collect important biological parameters facilitated by the isolation of novel viruses that classical virological techniques provide.

One resource that has the potential to leverage the power of high throughput sequencing and classical virology is historical virus isolate collections [3]. Sequencing of virus isolates held in historical collections can allow for the linkage of critical serological and biological data with genome sequences, allow for the identification of type isolates for viruses discovered in sequencing surveys, prevent redundancy and duplicated effort in the systematic characterisation of viruses, and allow for the identification of unknown viruses isolated in the past—potentially shedding light on historical epidemiological and disease events and allowing for improved prediction of, and responses to, future outbreaks.

A growing body of research is demonstrating a link between climate change, changes in trading patterns of animals and animal products, and the expansion in geographic ranges of several pathogenic disease threats [4]. In particular, the recent spread of formerly obscure arboviral pathogens such as Chikungunya and Zika virus highlights the importance of viral discovery and taxonomy in order to rapidly identify the etiological agents and mount responses to emergent disease outbreaks [5]. This is especially relevant to Australia, where a number of undifferentiated febrile illnesses go undiagnosed [6] and a number of poorly understood arboviruses (e.g., Edge Hill, Kokobera, Alfuy, Sinbis, and Stratford viruses) are known to cause human infection, but diagnostic tests are not readily available [7]. It stands to reason that: (1) a number of the undiagnosed febrile illnesses are caused by undiscovered arboviruses and (2) a considerable diversity of potentially human infectious arboviruses are yet to be discovered in the Australasian region.

Northern Australia has been a historical hotbed of virus discovery, with over 80 novel RNA viruses from 9 families and 16 genera reported from Australia and Papua New Guinea, largely driven by research efforts in Northern Australia [1]. A particular focus of viral diversity for Northern Australia has been arboviral taxa—often driven by outbreaks of both human and zoonotic encephalitis [1]. Unlike other arboviral hotspots such as the Amazon Basin and Southeast Asia, which tend to be endemic areas for widely distributed arboviruses of high clinical health and veterinary importance such as Zika, West Nile, Dengue, Chikungunya, and Yellow Fever [8,9], Northern Australia tends towards a higher number of unique arboviruses, thought to be due to the monsoonal wet–dry tropical climate that supports a range of distinct host and vector species [10,11]. A considerable proportion of these viruses remain insufficiently characterised with respect to their potential for human infectivity and subsequent risk of zoonotic spill-over [6].

Two of the most diverse arboviral genera in Northern Australia are *Orthobunyavirus* and *Orbivirus* [11]. *Orthobunyavirus* is a large genus (~170 species) of the family *Peribunyaviridae*, a family of negative sense, single stranded RNA viruses characterised by tripartite genomes and spherical, enveloped capsids [12]. They are transmitted by arthropod vectors, with amplification cycles in a diversity of vertebrate hosts. *Orthobunyaviruses* are largely transmitted by mosquitoes; however, some have been isolated from tabanoids [13], phlebotomines [14], ticks [15], and bedbugs [16]. A number of *Orthobunyaviruses* are known to cause human infections, including La Crosse virus in the United States [17], members of the Bunyamwera serogroup in sub-Saharan Africa and the Americas [18], and Oropuche orthobunyavirus in South America. Akabane serogroup *orthobunyaviruses* are a significant cause of livestock disease in Australasia, the Middle East and sub-Saharan Africa [19], and are vectored by biting midges of the genus Culicoides [20]. *Orthobunyaviruses* generally cause acute febrile illness and encephalitis when presenting in human infections [21]. The taxonomy of the *Orthobunyavirises* is complex and remains fluid as serotyping has led to incomplete characterisation of viruses within the genus [12].

The genus *Orbivirus* is within the Reoviridae family, comprising 22 serogroups and several unclassified isolates [22]. *Orbiviruses* have a double stranded RNA genome comprising 10 segments encapsulated by a non-enveloped, architecturally complex icosahedral capsid [23]. *Orbiviruses* are transmitted by ticks [24] and various haematophagus insect vectors (e.g., Culicoides midges [25], mosquitoes [26], and sandflies) and have a wide host range, including bovids, equids, camelids, marsupials, sloths, bats, birds, canines, felines, and humans [27]. There are three economically important animal diseases in the *Orbivirus* genus—Bluetongue virus, African horse sickness virus, and epizootic haemorrhagic disease virus [28]. They produce a spectrum of disease ranging from subclinical infection to high morbidity and mortality haemorrhagic fevers [29].

In the present study, we undertook high throughput sequencing of 76 virus isolates isolated between 1982 and 2004 for which serological testing at the time of isolation yielded uncertain results. The 76 isolates were selected based on serological ambiguity in conjunction with considerations of host range and geographic distribution, to ensure broad representation across potential sources of viral diversity. Isolates were held in the viral isolate collection at the Northern Territory government’s Berrimah Veterinary Laboratory and the Australian Centre for Disease Preparedness (ACDP).

## 2. Materials and Methods

### 2.1. Isolation of Viruses from Mosquitoes

Mosquitoes were trapped using encephalitis virus surveillance CO_2_ baited light traps [30]. Collections were transferred to the entomology laboratory for speciation and separation of gravid specimens. Mosquitoes were manually sorted into monospecific pools of not more than 50 individuals, and stored in liquid nitrogen prior to transfer to the virology laboratory for virus isolation and identification.

Isolation methods between 1981 and 1991 were as described [31]. Briefly, monospecific pools of mosquitoes were removed from an ultracold (−70 °C) freezer and homogenised in a chilled mortar and pestle containing a small quantity of sterile fine grain sand and 5 mL of brain heart infusion broth containing antibiotics penicillin (6 mg/mL), streptomycin (20 mg/mL) and amphotericin B (2.5 mg/mL). The resulting suspension was transferred to a 10 mL polypropylene centrifuge tube and held overnight at 4 °C. The following day the homogenates were clarified by centrifugation (2000× *g*, 4 °C, 15 min) and inoculated to two baby hamster kidney clone 21 (BHK-21) [32] cell cultures. This method was followed from 1981 to 1984. Between 1985 and 1992, two *Aedes albopictus* (C6/36) [33] stationary cell cultures growing in tubes were used as the first passage amplification step. First passage tubes were incubated for seven days before pooling and passage to two BHK-21 and two hamster lung (HmLu-1) [34] cell culture tubes. Second passage tubes were incubated for seven days at 37 °C before pooling and final passage to one BHK-21 and one HmLu-1. Cell culture tubes were inspected for the presence of cytopathic effect (CPE) from day three to day seven during the second and third passages.

In 1992, Kontes pellet pestles replaced mortars and pestles. A small quantity of lapidary grinding powder was added to each microcentrifuge tube, followed by 200 µL of brain heart infusion broth containing penicillin G (6 mg/mL), streptomycin sulphate (20 mg/mL), and amphotericin B (2.5 mg/mL). The tubes were returned to 4 °C and held prior to use. From one to 50 monospecific mosquitoes were added to each 2 mL tube containing brain heart infusion broth and lapidary grinding powder. The mosquitoes were homogenised using a plastic disposable pestle attached to either a Kontes grinding motor or a laboratory homogeniser. The homogenised mosquitoes were transferred to a 10 mL centrifuge tube containing 5 mL of brain heart infusion broth and antibiotics and held overnight at 4 °C (to restrict contamination of cell cultures) prior to clarification by centrifugation (2000× *g* for 15 min at 4 °C). Two confluent C6/36 cell culture tubes containing 2 mL of minimum essential medium and 10% foetal bovine serum and mosquito homogenate were incubated stationary at 25 °C for seven days. On day seven, tubes were inspected for fungal or bacterial contamination, and placed in an ultrasonic cleaning bath for 60 min. Each pair of C6/36 tubes was pooled together before passage to two BHK-21 and two HmLu-1 cell culture tubes. Contaminated cultures were processed last and filtered through a 0.22 µm sterile membrane filter (Sartorius Minisart Plus Cat. 178 23K or Millipore Millex-GP (Burlington, MA, USA)) before inoculation. The BHK-21 and HmLu-1 cultures were inspected for CPE from day three to day seven. At passage two on day seven, CPE negative cultures were pooled and inoculated to single BHK-21 and HmLu-1 cell culture tubes. Cultures were inspected for the presence of CPE for seven days. CPE positive cultures were removed, and CPE negative cultures were discarded. Seed virus stocks were prepared from all CPE positive cultures by inoculation of six tubes of BHK-21 or HmLu-1, or if both cell lines were CPE-positive, BHK-21 was used to prepare the seed virus stock. Seed virus stock CPE was allowed to progress to 80–90% before harvesting, pooling, stabilisation with 1% bovine serum albumin, and storage at 4 °C and −70 °C. Storage of some unidentified viruses, particularly *Orbiviruses*, proved superior at 4 °C.

### 2.2. Virus Isolation from Cattle Blood

During 1979, permanent sentinel cattle herds were established at Coastal Plains Research Station (lat. 12°39′ S, long. 131°20′ E), Tortilla Flats Research Farm (lat. 13°05′ S long. 131°14′ E) and Berrimah Research Farm (lat. 12°26′ S, long. 130°55′ E). The cattle were bled weekly for virus isolation and monthly for retrospective serology. Each year, the herds were replaced with animals that were seronegative to bluetongue, epizootic haemorrhagic disease, Palyam virus, bovine ephemeral fever, and Simbu group viruses. Total herd size and composition have varied considerably. Over the years, there are generally forty to fifty cattle comprising an equal number of cows and steers.

Each heparinised blood sample was inoculated to each of three embryonated chicken eggs for virus isolation as previously described [35,36]. Briefly, heparinised blood samples were chilled immediately upon collection and transported to the laboratory and held at 4 °C overnight. A 50 µL sample of blood was aspirated from the sealed tube and lysed with 450 µL of sterile distilled water. The lysate was used as inoculum from three-, nine-, to eleven-day-old embryonated chicken eggs. Each egg received a 100 µL intravenous inoculation of blood lysate using a 1 mL Tuberculin syringe and 29 ga needle. Embryos dying during the first 24 h were recorded and discarded. Embryos dying from 24 to 120 h post inoculation were recorded and held at 4 °C prior to harvesting. At 120 h post inoculation, all remaining embryos were euthanised by placing them at −20 °C for 30 min (eggs were not frozen). Embryos were aseptically harvested, and those receiving the same inoculum and dying at about the same time were pooled.

Embryos, minus the head, were then homogenised in brain heart infusion broth containing penicillin (5 mg), streptomycin (3 mg) and amphotericin B (12 µg). The resulting homogenate was transferred to a 10 mL polypropylene centrifuge tube and centrifuged at 2000× *g* for 10 min. All homogenates were held at 4 °C overnight prior to inoculation to cell culture tubes.

In 1993, a less labour-intensive method was derived from a method used by the University of Western Australia [37]. Duplicate wells of a 96 well microtitre plate containing C6/36 mosquito cell cultures were used as a first passage, followed by two mammalian cell culture passages each comprising six cell lines. In the first passage, 15 µL of each sample (egg or mosquito homogenate) was inoculated into duplicate wells containing C6/36 mosquito cell cultures (seeding rate 2 × 10^5^/mL) and 150 µL of minimum essential medium growth medium in a 96 well flat bottomed microtitre plate. Plates were incubated at ambient room temperature (25 °C) in a humidified container for seven days. In the second passage, 15 µL of first passage supernatant was inoculated to identical wells of seven plates, each containing a different cell type. The wells of column A1-H1 were mixed, and 15 µL was transferred to the duplicate column A2-H2, mixed, and the procedure reversed. On completion of mixing, 15 µL was transferred to identical rows in each of seven cell culture plates, each containing a single cell type as follows: (1) BSR clone of BHK-21 cells [38] grown in basal medium Eagle’s growth medium with Earle’s salts, supplemented with 10% foetal bovine serum. (2) BHK 21 cells grown in basal Eagle’s growth medium. (3) Hamster lung cells grown in minimum essential medium growth medium. (4) Vero cells derived from African green monkey kidney cells grown in Medium 199 supplemented with 10% foetal bovine serum (Medium 199 growth medium). (5) Porcine stable equine kidney cells grown in minimum essential medium growth medium. (6) Calf pulmonary artery endothelial cells [39] grown in minimum essential medium growth medium. (7) C6/36 cells grown in minimum essential medium growth medium. At 80–100% CPE, the supernatant was aseptically removed from the CPE positive wells and inoculated to 25 cm^2^ tissue culture flasks for the production of seed stock virus. Inoculation of the third cell culture passage used the C6/36 s passage plates.

Each supernatant (150 or 300 µL) from CPE positive well(s) was aseptically removed from the infected wells and diluted 1:100. The diluted supernatant (virus) was then adsorbed in a BSR confluent 25 cm^2^ tissue culture flask containing 3 mL of basal medium Eagle’s supplemented with 5% heat inactivated foetal bovine serum for one hour. A further 8 mL of basal medium Eagle’s maintenance medium was added to the flask following adsorption. Generally, CPE was present in BSR cells, and this was the cell line of choice for the production of seed stock virus. Tissue culture supernatant was removed at 90% CPE, stabilised using 1% bovine serum albumin, aliquoted, and stored at −70 °C.

### 2.3. Virus Identification: Serogrouping of Virus Isolates

Mosquito viruses isolated from 1982 to 1992 were initially screened in a plaque reduction neutralisation test [40]. Briefly, 24 well cluster plates containing BHK-21 or BSR monolayers were overlayed with 250 μL of a homologous antibody of known titer in 4 wells down the plate. Each column contained a different antibody, and 4 viruses could be screened in a single plate. A single virus was inoculated per column and allowed to incubate with the antibody for 1–2 h. After the incubation period, a 2% agarose solution was added and mixed thoroughly and allowed to cool. Polyvalent hyperimmune antibodies prepared in rabbits to members of the bovine ephemeral fever, bluetongue, epizootic haemorrhagic disease, Palyam, Simbu, alphavirus, and flavivirus virus groups were used in a grouping plaque reduction neutralisation test. Isolates not neutralised were then screened against a panel of antibodies using a modification of the indirect fluorescent antibody method described by [41,42].

Further serology to group isolates using a comprehensive IFA panel—a panel of antisera provided by Charlie Calisher from CDC Fort Collins—allowed us to test for ~400 agents. Preliminary identification of virus isolates was conducted using serological grouping assays applied to cultured virus, rather than to host sera. All virus isolates were grown as adherent monolayers in tissue culture flasks; cells were only briefly placed in suspension during trypsinisation prior to preparation of cell suspensions for the IFA. Isolates were screened by plaque reduction neutralisation tests (PRNT) against panels of polyclonal hyperimmune antisera [40] targeting major arbovirus groups (e.g., flaviviruses, alphaviruses, orbiviruses). Isolates not neutralised were further screened using an indirect immunofluorescent antibody assay (IFA) with a comprehensive reference antisera panel [43]. In this context, serological assays were used to assess antigenic relationships between isolates and reference viruses, providing a first level of taxonomic classification prior to sequencing.

### 2.4. Virus Identification: Indirect Immunofluorescent Assay

A 25 cm^2^ tissue culture flask was used for each unidentified virus and infected with 100 TCID50. Cytopathic effect was allowed to progress to 50%. At 50% (2+) CPE, supernatant was removed, and the cells remaining in the flask were trypsinized from the flask and combined with the decanted supernatant. Trypsinising the remaining cells from the flask had no significant effect on the outcome of the indirect fluorescent antibody test. It did, however, save a considerable amount of time (days) waiting for cells to slough off the flask. The suspension was centrifuged at 2000× *g* for 15 min at 4 °C, and the pellet was resuspended in 4 mL of phosphate buffered saline containing 5% foetal bovine serum. Spot slides were prepared essentially as described by [42]. Briefly, 30 µL of cell suspension was added to each spot of ten, twelve spot-slides. The slides were air-dried and then immersed and fixed in cold acetone for 15 min. The slides were removed from acetone and air-dried prior to rinsing in phosphate buffered saline (to remove crystalline deposits) and acetone (to aid drying) before storage at −20 °C. It was realised that trypsinisation can alter or remove antigens of some viruses. Alternatively, if CPE was allowed to continue until the majority of cells were dislodged, identification of some viruses becomes difficult as the antigen concentration falls rapidly once CPE is advanced. Comparisons were made, and this method was determined to be a good compromise.

Group- or type-specific antibody (List 1) was diluted 1:25 and added to a prerecorded spot on each slide for each unidentified virus. All slides were incubated in a humidified container (150 mm disposable Petri dishes containing damp paper towels) at 37 °C for 1 h. After incubation, the slides were rinsed in phosphate buffered saline and then washed for 15 min in phosphate buffered saline. The slides were air dried and 30 µL of reconstituted anti-species fluorescein isothiocyanate conjugate (goat-anti-mouse fluorescein isothiocyanate, Cappel Cat. 55496) containing 0.4% trypan blue was added to each spot. The slides were returned to the humidified container and placed at 37 °C for 1 h. After incubation the slides were washed as previously described, and were not allowed to dry. A bead (~100 µL) of Aquamount mountant (BDH Cat. 36086) was laid in a strip down the centre of each wet slide, and a large coverslip (60 × 24 mm) was placed over the slide and air bubbles removed.

Fluorescence was visualised using an Olympus fluorescent microscope and subjectively estimated from 0 (negative) to 4 + (strong positive) and recorded for each spot. Spots recorded as 2+ or greater were considered positive.

### 2.5. Sequencing and Bioinformatics Analysis

RNA was extracted using an RNeasy Plus Mini Kit (Qiagen, Gernamy). Library preparation was undertaken using the Illumina Stranded Total RNA Prep, Ligation with Ribo-Zero Plus kit as per the manufacturer’s recommendations. Isolates were sequenced at the Australian Centre for Disease Preparedness (ACDP) using an Illumina NextSeq2000 platform. Sequences were adapter trimmed and quality filtered using Trimmomatic v0.4.0 [44] and assembled using SPAdes v3.15.5 [45] at k = 31, 55, 75, 95, and 127 and otherwise default settings. Contigs were compared with the Rdrp-scan database [46] using the Blastx function of Diamond v2.1.8.162 [47]. Genomes with significant hits (e > 10^−5^) were used as references for alignment using BWA [48] on trimmed reads for confirmation of sample identity.

Alignments of the Sedoreoviriade RdRp gene [22] and Peribunyaviridae L segment sequence [12] were downloaded from the International Committee on the Taxonomy of Viruses (ICTV) website. Consensus fasta files of aligned viral isolates were produced using Samtools [49], and an alignment of samples generated in this study was aligned with the datasets downloaded from ICTV using MAFFT [50] using the E-INS-I algorithm. The most appropriate substitution model for the Sederoreoviridae and Peribunyaviridae alignments was determined using jModelTest [51], and phylogenies were generated using MrBayes v3.2.7 [52] using 4 heated chains and 1,100,000 generations sampled every 200 generations. The first 10% of trees were discarded as burn in and run parameters were evaluated using Tracer v1.7.1 [53].

## 3. Results

For many of the isolates, inadequate nucleic acid could be recovered to perform effective sequencing. Resultantly, we obtained de novo assemblies and RdRp blast matches for 43 of the 76 isolates initially selected for sequencing (Table 1). In these, we had provisional serotype identifications for 30 isolates. Viruses were isolated between 1982 and 2004, with two samples isolated from *Aedes lineatopennis*, three from *Aedes (Och) normanensis*, one from *Anopheles annulipes*, two from Anopheles farauti, two from Anopheles amictus, six from *Anopheles meraukensis*, and nine from *Culex annulirostris*. Two samples were isolated from pooled mosquitoes and two from unnamed insects. The remainder were isolated from mammalian hosts, including ten from *Bos indicus*, one from *Bubalus bubalis*, two from *Equus caballus*, and one from *Osphranter rufus*. Samples were collected primarily from regions within the Northern Territory of Australia, including Darwin, Katherine, Kakadu National Park, Jabiru, Nganmarriyanga (Palumpa), Larrimah, Mataranka, and Beatrice Hill Farm (Coastal Plains Research Centre). The two unnamed insect samples were collected in Western Australia.

**Figure 1:**
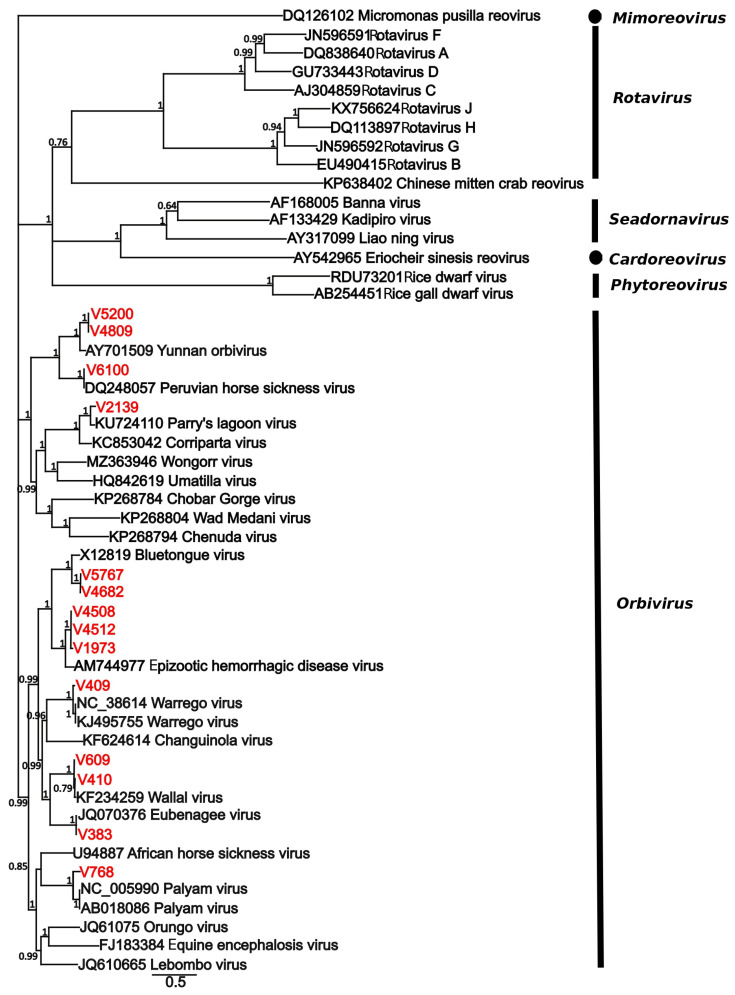
Phylogenetic tree of the RdRp gene placing isolates sequenced in this study within the *Sedereoviridae*. Sequences from this study are shown in red, and the tip label corresponds to the virus number in Table 1. Sequences were aligned with MAFFT, and the tree was estimated using MrBayes v3.2.7. Numbers at nodes indicates posterior probability. The scale bar represents substitutions/site.

**Figure 2:**
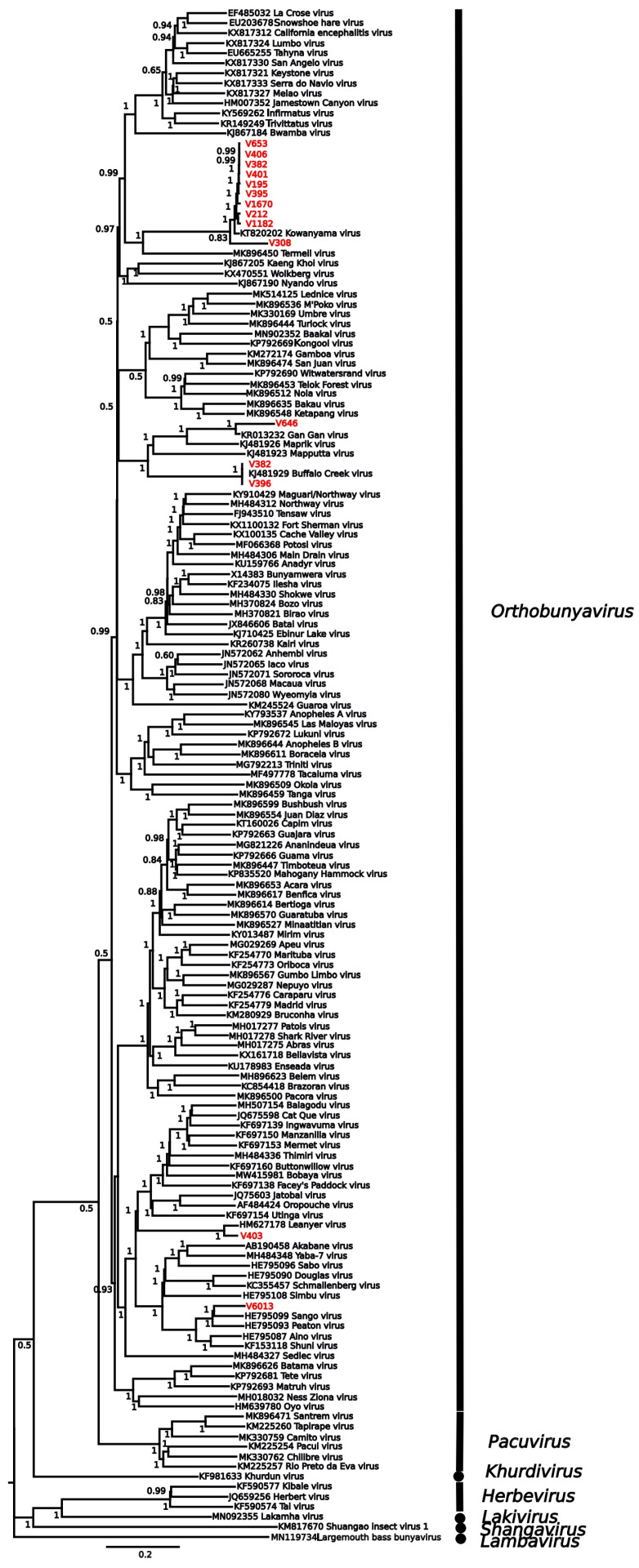
Phylogenetic tree of the L segment placing isolates sequenced in this study within the Peribunyaviridae. Sequences from this study are shown in red, and the tip label corresponds to the virus number in Table 1. Sequences were aligned with MAFFT, and the tree was estimated using MrBayes v3.2.7. Numbers at nodes indicate posterior probability. The scale bar represents substitutions/site.

All viruses were matched to at least 91% similarity with RdRp genes from known viruses (Table 1). Isolates included one > 99 match to Hapavirus Holmes (*Rhabdovirivdae/Alpharhabdoviridae*), 23 isolates were matched to eleven species in the genus *Orbivirus* (*Reovirales/Sedoreoviridae*), and 19 to six species in the genus *Orthobunyavirus* (*Bunyavirales/Peribunyaviridae*). Phylogenetic analysis of both the *Sedoreoviridae* (Figure 1) and *Peribunyaviridae* (Figure 2) confirmed Blastx results both with respect to species identification and the percentage match with respect to topology and branch lengths.

## 4. Discussion

All the 43 virus isolates that we were able to generate sequence data that was adequate for identification via RdRp Blast comparison were closely related (between 91 and >99% sequence identity) to known viral taxa (Table 1). Most of these identifications were confirmatory with provisional serological identifications, with the exception of an isolate serotypically identified as Bluetongue virus and sequenced as Epizootic haemorrhagic disease virus. It is not necessarily surprising that the taxa identified in this study are not completely novel, as the process of isolation and amplification is likely to select for a specific subset of viruses already characterised. However, the data does add considerably to the body of genomic data for several relatively understudied species, and potentially sheds light on novel serotypes and genotypes within species groups. The relatively low recovery rate (43/76 isolates) reflects variability in nucleic acid integrity across samples. Future studies may benefit from unbiased enrichment approaches (e.g., rRNA depletion, host subtraction, random-primed amplification) alongside optimised extraction and preservation methods to increase recovery from poorly preserved or low-titre material.

Species delimitation is a field fraught with challenges, as taxonomy places discrete boundaries on evolutionary processes that operate on continuums [54]. While some describe viral taxonomy as a categorisation of convenience, there is practical utility to taxonomic nomenclature for researchers in the field [54,55]. There are varying levels of genetic divergence between lineages considered distinct species, and frequently significant divergences within species. In this study, most isolates are likely members of existing species based on genetic evidence; however, some are significantly divergent from their closest match. However, due to incomplete genome sequencing, the provided virus classifications are tentative. For example, V1664, V197, V3265, V3289, and V6250 are all ~5% divergent from Wongorr virus, and V409 is 9% divergent from Warrego virus based on Blastx matches, but recovery of the RdRp gene was not of sufficient coverage for phylogenetic placement. This could be driven by inadequate sequencing depth but is also possibly due to poor mapping to the reference sequence due to divergence from it. As the identity of the isolates was unknown prior to sequencing, specific sequence-targeted amplification could not be performed. Although additional rounds of enrichment could have increased genome coverage per isolate, our study focused on maximising virus discovery across the collection rather than achieving complete genome sequences for each isolate. V6013 was a 95% match to Sango virus based on Blastx results, but alignment of the RdRp L segment gene using MAFFT reveals a pairwise identity of only 80.7%. By comparison, pairwise identity of Sango virus to its nearest relative—Peaton virus—is 84.7%. Based purely on genetic identity, it would prima facie appear that V6013 is a novel taxon; however, follow up evidence from other life history criteria is necessary to support this. This study shows, at least putatively, that multiple novel species of virus may be preliminarily identified by sequencing historical virus isolate collections, considerably narrowing down the isolates worthy of further investigation for the discovery of novel taxa.

Hapaviruses form a monophyletic group within the Alpharhabdovirinae subfamily of the Rhaboviridae, within a larger clade of arthropod-borne rhabdovirsues. They have been primarily isolated from culicine mosquitoes and passerine birds [56], although there is evidence for Hapavirus antibodies in marsupials [56], cattle [57], and a hospitalised human patient [58]. Pairwise alignment of the whole genome sequence recovered from Sample V1163 has a 99.9% pairwise nucleotide identity with the Hapavirus holmes genome, and a 76.4% pairwise identity with the nearest relative—Wongabel virus [58], strongly indicating that the virus isolate sequenced in this study is Hapavirus holmes. The Hapavirus genus was recently established in 2017 [59], and relatively little is known about the life history and gene function of the group [58]. Another complete genome from this enigmatic group of arboviruses presents a resource in generating a more complete understanding of the group.

Orbiviruses are highly prevalent in Northern Australia [60], and a number are emergent zoonotic pathogens (e.g., Corriparta virus [61], Lebombo virus [62], Orungo virus [63]) or have recently spilled over into commercially important livestock species, e.g., Peruvian horse sickness virus and Yunnan virus [64]. Similarly, orthobunyaviruses are especially diverse in Northern Australia, and responsible for a wide spectrum of zoonotic disease—especially species from the Bunyamwera [65], California [66], and Simbu [67] serocomplexes. The impacts of human orthobunyaviral infections can be significant; e.g., 10% of paediatric infections of La Crosse virus result in long-term cognitive sequelae [68], and Oropouche virus causes symptomology similar to Zika and Dengue viruses in South America [69]. Given the high proportion of undiagnosed febrile illnesses in northern Australia [6], it is likely that many of these cases are caused by either novel or poorly characterised Orbivirus and Orthobunyavirus taxa. The role of novel arboviral etiological agents in infections and their role in febrile illnesses in non-specific fevers in humans is of significant public health interest [6,70], and due to the increase in factors associated with novel epizootic arboviral pathogens [71,72], will be of continued importance. As this study demonstrates, historical viral collections can allow for the pre-emptive characterisation of novel orbivurses, providing an invaluable resource for disease outbreak preparedness and can provide a critical tool for the development of both diagnostic and therapeutic agents for the treatments of these diseases.

## Figures and Tables

**Table 1:** Summary of isolates for which sequence data was generated. The virus number is the identification number in the Berrimah Veterinary Laboratory collection. Provisional ID was based upon serological results obtained at the time of isolation. Sequenced ID result refers to the sequence ID with the best match in the RdRp scan database as determined by Blast X comparison. Match % is the pairwise identity to the RDRp gene, and % Coverage is the percentage of the reference genome identified in an assembly of the sample. CPRS—Coastal Plains Research Station.

Virus	Location	Date	Host Species	Provisional ID	Best Match			
					Name	GenBank ID	% Match	% Coverage
V1163	Darwin	3 February 1987	*C. annulirostris*	rhabdovirus-like virus	Hapavirus holmes	ASM90779	>99	93.69
V1182	Palumpa	15 April 1987	*A. amictus*	Bunyavirus-like virus	Kowanyama virus	AMR73391	>99	100
V1664	Katherine	8 February 1989	*A. lineatopennis*	Wongorr virus	Wongorr virus	YP009665177	95	28.8
V1670	Katherine	8 February 1989	*C. annulirostris*	orbivirus-like virus	Kowanyama virus	AMR73391	>99	99.81
V178	Darwin	4 March 1982	*C. annulirostris*	Wongorr virus	Wongorr virus	YP009665177	93	23.07
V193	Darwin	28 April 1982	*A. meraukensis*	Bunyavirus	Kowanyama virus	AMR73391	>99	100%
V197	Darwin	16 March 1982	*A. farauti*	Wongorr virus	Wongorr virus	YP009665177	95	16.59
V1973	Darwin rural	21 February 1990	*C. annulirostris*	Bluetongue virus	Epizootic hemorrhagic disease virus	CAN99549	>99	17.5
V212	Darwin	29 March 1982	*A. meraukensis*	Bunyavirus-like virus	Kowanyama virus	AMR73391	>99	99.78
V213	Darwin	20 March 1982	*A. farauti*	Mapputta virus	Mapputta orthobunyavirus	AKO90170	98	100
V2139	Kakadu	8 May 1991	*C. annulirostris*	orbivirus-like virus	Parry’s Lagoon virus	ANH10670	97	99.79
V308	Unknown	1 August 1983	Mosquito pool	Unknown	Kowanyama virus	AMR73391	>99	68.53
V3265	Western Australia	19 October 1994	Unnamed insect	Wongorr virus	Wongorr virus	YP009665177	95	26.55
V3289	Western Australia	21 November 1994	Unnamed insect	Wongorr virus	Wongorr virus	YP009665177	95	22.44
V382	Darwin	29 March 1983	*A. meraukensis*	Bunyavirus-like virus	Buffalo Creek Orthobunyavirus	AJD77610	>99	100
V383	Katherine	29 March 1983	*A. lineatopennis*	Eubenangee virus	Eubenangee virus	YP009507706	>99	99.8
V3904	CPRS	27 June 1996	*B. indicus*	Unknown	Yunnan orbivirus	YP443925	97.6	27.67
V395	Katherine	30 April 1983	*A. amictus*	Unknown	Kowanyama virus	AMR73391	>99	97.91
V396	Darwin	4 May 1983	*A. annulipes*	Bunyavirus-like virus	Buffalo Creek Orthobunyavirus	AJD77610	>99	100
V400	Darwin	27 April 1983	*A. meraukensis*	Bunyavirus-like virus	Kowanyama virus	AMR73391	>99	99.97
V401	Unknown	1 August 1983	Mosquito pool	Unknown	Kowanyama virus	AMR73391	>99	99.8
V403	Darwin	17 May 1983	*A. meraukensis*	Bunyavirus	Leanyer Orthobunyavirus	AEA02985	>99	99.96%
V406	Darwin	17 May 1983	*A. meraukensis*	Bunyavirus	Kowanyama virus	AMR73391	>99	99.86%
V409	Jabiru	3 June 1983	*C. annulirostris*	Warrego virus	Warrego virus	AGX86082	91	99.65%
V410	Jabiru	03 June 1983	*C. annulirostris*	Wallal virus	Wallal virus	AGX00987	>99	99.29%
V4135	CPRS	9 January 1996	*B. indicus*	Unknown	Kowanyama virus	AMR73391	>99	
V4330	CPRS	23 June 1997	*B. bubalis*	Epizootic hemorrhagic disease virus	Epizootic hemorrhagic disease virus	CAN99549	>99	99.94%
V4508	CPRS	19 November 1998	*B. indicus*	Unknown	Epizootic hemorrhagic disease virus	CAN99549	>99	18.80%
V4512	CPRS	8 January 1999	*B. indicus*	Epizootic hemorrhagic disease virus	Epizootic hemorrhagic disease virus	CAN99549	>99	15.93%
V4682	CPRS	17 June 1999	*B. indicus*	Unknown	Bluetongue virus	AKV60532	>99	34.46%
V4809	CPRS	10 February 2000	*B. indicus*	Unknown	Yunnan orbivirus	YP443925	96.1	43.65%
V4835	Katherine	N/A	*E. caballus*	Unknown	Peruvian horse sickness virus	YP460038	>99	60.00%
V5200	CPRS	5 April 2001	*B. indicus*	Unknown	Yunnan orbivirus	YP443925	96.5	25.22%
V5767	CPRS	5 December 2002	*B. indicus*	Bluetongue virus	Bluetongue virus	AKV60532	>99	57.61%
V6013	CPRS	28 August 2003	*B. indicus*	Unknown	Sango orthobunyavirus	YP009666881	95	95.05%
V609	Larrimah	16 February 1984	*A. normanensis*	Wallal virus	Wallal virus	AGX00987	>99	99.47%
V6100	Katherine	5 February 2004	*E. caballus*	Unknown	Peruvian horse sickness virus	YP460038	>99	99.30%
V6228	CPRS	22 April 2004	*B. indicus*	Unknown	Yunnan orbivirus	YP443925	97.4	6.29%
V6250	TWP	27 May 2004	*O. rufus*	Wongorr virus	Wongorr virus	YP009665177	95	25.65%
V646	Mataranka	15 March 84	*A. normanensis*	Bunyavirus-like virus	Gan Gan orthobunyavirus	ALQ43836	>99	55.73%
V653	Mataranka	11 April 1984	*A. normanensis*	Orbivirus-like virus	Kowanyama virus	AMR73391	94	99.97
V768	Darwin	30 January 1985	*C. annulirostris*	Palyam virus	Palyam virus	QCU80060	98.5	98.02%
V771	Darwin	12 February 1985	*C. annulirostris*	Wongorr virus	Kowanyama virus	AMR73391	>99	99.99

## Data Availability

Sequencing data is submitted to NBCI under accession SUB15364976.

## References

[1] Vasilakis N., Tesh R.B., Popov V.L., Widen S.G., Wood T.G., Forrester N.L., Gonzalez J.P., Saluzzo J.F., Alkhovsky S., Lam S.K. (2019). Exploiting the Legacy of the Arbovirus Hunters. Viruses.

[2] Next Generation Sequencing Technologies for Insect Virus Discovery—PubMed. https://pubmed.ncbi.nlm.nih.gov/22069519/.

[3] Jones R.A.C., Boonham N., Adams I.P., Fox A. (2021). Historical Virus Isolate Collections: An Invaluable Resource Connecting Plant Virology’s Pre-Sequencing and Post-Sequencing Eras. Plant Pathol..

[4] Petersen L.R., Holcomb K., Beard C.B. (2022). Climate Change and Vector-Borne Disease in North America and Europe. J. Health Monit..

[5] Huang Y.-J.S., Higgs S., Vanlandingham D.L. (2019). Emergence and Re-Emergence of Mosquito-Borne Arboviruses. Curr. Opin. Virol..

[6] Gyawali N., Bradbury R.S., Aaskov J.G., Taylor-Robinson A.W. (2017). Neglected Australian Arboviruses and Undifferentiated Febrile Illness: Addressing Public Health Challenges Arising From the ‘Developing Northern Australia’ Government Policy. Front. Microbiol..

[7] Gyawali N., Bradbury R.S., Aaskov J.G., Taylor-Robinson A.W. (2017). Neglected Australian Arboviruses: Quam Gravis?. Microbes Infect..

[8] Gould E.A., Solomon T. (2008). Pathogenic Flaviviruses. Lancet.

[9] Messina J.P., Brady O.J., Scott T.W., Zou C., Pigott D.M., Duda K.A., Bhatt S., Katzelnick L., Howes R.E., Battle K.E. (2014). Global Spread of Dengue Virus Types: Mapping the 70 Year History. Trends Microbiol..

[10] Taylor-Robinson A.W. (2024). Complex Transmission Epidemiology of Neglected Australian Arboviruses: Diverse Non-Human Vertebrate Hosts and Competent Arthropod Invertebrate Vectors. Front. Microbiol..

[11] Huang B., Allcock R., Warrilow D. (2016). Newly Characterized Arboviruses of Northern Australia. Virol. Rep..

[12] Hughes H.R., Adkins S., Alkhovskiy S., Beer M., Blair C., Calisher C.H., Drebot M., Lambert A.J., de Souza W.M., Marklewitz M. (2020). ICTV Virus Taxonomy Profile: Peribunyaviridae. J. Gen. Virol..

[13] Isolations of Jamestown Canyon Virus (Bunyaviridae: Orthobunyavirus) from Field-Collected Mosquitoes (Diptera: Culicidae) in Connecticut, USA: A Ten-Year Analysis, 1997–2006—PubMed. https://pubmed.ncbi.nlm.nih.gov/18386967/.

[14] Labuda M. (1991). Arthropod Vectors in the Evolution of Bunyaviruses. Acta Virol..

[15] Lasecka L., Baron M.D. (2014). The Molecular Biology of Nairoviruses, an Emerging Group of Tick-Borne Arboviruses. Arch. Virol..

[16] Williams J.E., Imlarp S., Top F.H., Cavanaugh D.C., Russell P.K. (1976). Kaeng Khoi Virus from Naturally Infected Bedbugs (Cimicidae) and Immature Free-Tailed Bats. Bull. World Health Organ..

[17] Henderson B.E., Coleman P.H. (1971). The Growing Importance of California Arboviruses in the Etiology of Human Disease. Prog. Med. Virol..

[18] Dutuze M.F., Nzayirambaho M., Mores C.N., Christofferson R.C. (2018). A Review of Bunyamwera, Batai, and Ngari Viruses: Understudied Orthobunyaviruses with Potential One Health Implications. Front. Vet. Sci..

[19] Hayama Y., Moriguchi S., Yanase T., Ishikura Y., Abe S., Higashi T., Ishikawa H., Yamamoto T., Kobayashi S., Murai K. (2016). Spatial Epidemiological Analysis of Bovine Encephalomyelitis Outbreaks Caused by Akabane Virus Infection in Western Japan in 2011. Trop. Anim. Health Prod..

[20] Jennings M., Mellor P.S. (1989). *Culicoides*: Biological Vectors of Akabane Virus. Vet. Microbiol..

[21] Weidmann M., Rudaz V., Nunes M.R.T., Vasconcelos P.F.C., Hufert F.T. (2003). Rapid Detection of Human Pathogenic Orthobunyaviruses. J. Clin. Microbiol..

[22] Matthijnssens J., Attoui H., Bányai K., Brussaard C.P.D., Danthi P., del Vas M., Dermody T.S., Duncan R., Fāng Q., Johne R. (2022). ICTV Virus Taxonomy Profile: Sedoreoviridae 2022. J. Gen. Virol..

[23] Roy P. (1996). Orbivirus Structure and Assembly. Virology.

[24] Belaganahalli M.N., Maan S., Maan N.S., Brownlie J., Tesh R., Attoui H., Mertens P.P.C. (2015). Genetic Characterization of the Tick-Borne Orbiviruses. Viruses.

[25] Calisher C.H., Mertens P.P.C., Mellor P.S., Baylis M., Hamblin C., Mertens P.P.C., Calisher C.H. (1998). Taxonomy of African Horse Sickness Viruses. African Horse Sickness.

[26] Liehne P.F., Anderson S., Stanley N.F., Liehne C.G., Wright A.E., Chan K.H., Leivers S., Britten D.K., Hamilton N.P. (1981). Isolation of Murray Valley Encephalitis Virus and Other Arboviruses in the Ord River Valley 1972-1976. Aust. J. Exp. Biol. Med. Sci..

[27] Maan S., Belaganahalli M.N., Maan N.S., Attoui H., Mertens P.P.C., Malik Y.S., Singh R.K., Yadav M.P. (2020). Orbiviruses. Emerging and Transboundary Animal Viruses.

[28] Maclachlan N.J., Guthrie A.J. (2010). Re-Emergence of Bluetongue, African Horse Sickness, and Other Orbivirus Diseases. Vet. Res..

[29] DeBiasi R.L., Tyler K.L. (2015). Orthoreoviruses and Orbiviruses. Mandell, Douglas, and Bennett’s Principles and Practice of Infectious Diseases.

[30] Rohe D.L., Fall R.P. (1979). A Miniature Battery Powered CO2 Baited Light Trap for Mosquito Borne Encephalitis Surveillance. Bull. Soc. Vector Ecol..

[31] Whelan P.I., Weir R. (1993). The Isolation of Alpha and Flavi Viruses from Mosquitoes in the Northern Territory 1982–1992.

[32] Macpherson I., Stoker M. (1962). Polyoma Transformation of Hamster Cell Clones—An Investigation of Genetic Factors Affecting Cell Competence. Virology.

[33] Igarashi A. (1978). Isolation of a Singh’s Aedes Albopictus Cell Clone Sensitive to Dengue and Chikungunya Viruses. J. Gen. Virol..

[34] Hsu T.C., Zenzes M.T. (1964). Mammalian Chromosomes in Vitro. XVII. Idiogram Chin. Hamster. J. Natl. Cancer Inst..

[35] Gard G.P., Shorthose J.E., Weir R.P., Erasmus B.J. (1987). The Isolation of a Bluetongue Serotype New to Austrlia. Aust. Vet. J..

[36] Goldsmit L., Barzilai E. (1968). An Improved Method for the Isolation and Identification of Bluetongue Virus by Intravenous Inoculation of Embryonating Chicken Eggs. J. Comp. Pathol..

[37] Lindsay M.D., Broom A.K., Wright A.E., Johansen C.A., Mackenzie J.S. (1993). Ross River Virus Isolations from Mosquitoes in Arid Regions of Western Australia: Implication of Vertical Transmission as a Means of Persistence of the Virus. Am. J. Trop. Med. Hyg..

[38] Sato M., Maeda N., Yoshida H., Urade M., Saito S., Miyazaki T., Shibata T., Watanabe M. (1977). Plaque Formation of Herpes Virus Hominis Type 2 and Rubella Virus in Variants Isolated from the Colonies of BHK21/WI-2 Cells Formed in Soft Agar. Arch. Virol..

[39] Del Vecchio P.J., Smith J.R. (1981). Expression of Angiotensin-Converting Enzyme Activity in Cultured Pulmonary Artery Endothelial Cells. J. Cell. Physiol..

[40] Gard G.P., Kirkland P.D. (1993). Bluetongue: Virology and Serology. https://www.cabidigitallibrary.org/doi/full/10.5555/19932290397.

[41] Zeller H.G., Karabatsos N., Calisher C.H., Digoutte J.-P., Cropp C.B., Murphy F.A., Shope R.E. (1989). Electron microscopic and antigenic studies of uncharacterized viruses. II. Evidence suggesting the placement of viruses in the familyBunyaviridae. Arch. Virol..

[42] Zeller H.G., Karabatsos N., Calisher C.H., Digoutte J.P., Cropp C.B., Murphy F.A., Shope R.E. (1989). Electron mi-croscopic and antigenic studies of uncharacterized viruses. III. Evidence suggesting the placement of viruses in the family Reoviridae. Arch. Virol..

[43] Calisher C.H. (1994). Medically important arboviruses of the United States and Canada. Clin. Microbiol. Rev..

[44] Bolger A.M., Lohse M., Usadel B. (2014). Trimmomatic: A Flexible Trimmer for Illumina Sequence Data. Bioinformatics.

[45] Bankevich A., Nurk S., Antipov D., Gurevich A.A., Dvorkin M., Kulikov A.S., Lesin V.M., Nikolenko S.I., Pham S., Prjibelski A.D. (2012). SPAdes: A New Genome Assembly Algorithm and Its Applications to Single-Cell Sequencing. J. Comput. Biol..

[46] Charon J., Buchmann J.P., Sadiq S., Holmes E.C. (2022). RdRp-Scan: A Bioinformatic Resource to Identify and Annotate Divergent RNA Viruses in Metagenomic Sequence Data. Virus Evol..

[47] Buchfink B., Reuter K., Drost H.-G. (2021). Sensitive Protein Alignments at Tree-of-Life Scale Using DIAMOND. Nat. Methods.

[48] Li H. (2013). Aligning Sequence Reads, Clone Sequences and Assembly Contigs with BWA-MEM. arXiv.

[49] Danecek P., Bonfield J.K., Liddle J., Marshall J., Ohan V., Pollard M.O., Whitwham A., Keane T., McCarthy S.A., Davies R.M. (2021). Twelve Years of SAMtools and BCFtools. GigaScience.

[50] Katoh K., Misawa K., Kuma K., Miyata T. (2002). MAFFT: A Novel Method for Rapid Multiple Sequence Alignment Based on Fast Fourier Transform. Nucleic Acids Res..

[51] Darriba D., Taboada G.L., Doallo R., Posada D. (2012). jModelTest 2: More Models, New Heuristics and Parallel Computing. Nat. Methods.

[52] Ronquist F., Teslenko M., van der Mark P., Ayres D.L., Darling A., Höhna S., Larget B., Liu L., Suchard M.A., Huelsenbeck J.P. (2012). MrBayes 3.2: Efficient Bayesian Phylogenetic Inference and Model Choice across a Large Model Space. Syst. Biol..

[53] Rambaut A., Drummond A.J., Xie D., Baele G., Suchard M.A. (2018). Posterior Summarization in Bayesian Phylogenetics Using Tracer 1.7. Syst. Biol..

[54] Peterson A.T. (2014). Defining Viral Species: Making Taxonomy Useful. Virol. J..

[55] Bobay L.-M., Ochman H. (2018). Biological Species in the Viral World. Proc. Natl. Acad. Sci. USA.

[56] Gubala A., Davis S., Weir R., Melville L., Cowled C., Walker P., Boyle D. (2010). Ngaingan Virus, a Macropod-Associated Rhabdovirus, Contains a Second Glycoprotein Gene and Seven Novel Open Reading Frames. Virology.

[57] Doherty R.L., Carley J.G., Standfast H.A., Dyce A.L., Kay B.H., Snowdon W.A. (1973). Isolation of Arboviruses from Mosquitoes, Biting Midges, Sandflies and Vertebrates Collected in Queensland, 1969 and 1970. Trans. R. Soc. Trop. Med. Hyg..

[58] Gubala A., Walsh S., McAllister J., Weir R., Davis S., Melville L., Mitchell I., Bulach D., Gauci P., Skvortsov A. (2017). Identification of Very Small Open Reading Frames in the Genomes of Holmes Jungle Virus, Ord River Virus, and Wongabel Virus of the Genus Hapavirus, Family Rhabdoviridae. Evol. Bioinform. Online.

[59] Amarasinghe G.K., Bào Y., Basler C.F., Bavari S., Beer M., Bejerman N., Blasdell K.R., Bochnowski A., Briese T., Bukreyev A. (2017). Taxonomy of the Order Mononegavirales: Update 2017. Arch. Virol..

[60] Cowled C., Melville L., Weir R., Walsh S., Hyatt A., Van Driel R., Davis S., Gubala A., Boyle D. (2007). Genetic and Epidemiological Characterization of Middle Point Orbivirus, a Novel Virus Isolated from Sentinel Cattle in Northern Australia. J. Gen. Virol..

[61] Boughton C.R., Hawkes R.A., Naim H.M. (1990). Arbovirus Infection in Humans in NSW: Seroprevalence and Pathogenicity of Certain Australian Bunyaviruses. Aust. N. Z. J. Med..

[62] Moore D.L., Causey O.R., Carey D.E., Reddy S., Cooke A.R., Akinkugbe F.M., David-West T.S., Kemp G.E. (1975). Arthropod-Borne Viral Infections of Man in Nigeria, 1964–1970. Ann. Trop. Med. Parasitol..

[63] Tomori O., Fabiyi A. (1976). Neutralizing Antibodies to Orungo Virus in Man and Animals in Nigeria. Trop. Geogr. Med..

[64] Attoui H., Mendez-lopez M.R., Rao S., Hurtado-Alendes A., Lizaraso-Caparo F., Mohd Jaafar F., Samuel A.R., Belhouchet M., Pritchard L.I., Melville L. (2009). Peruvian Horse Sickness Virus and Yunnan Orbivirus, Isolated from Vertebrates and Mosquitoes in Peru and Australia. Virology.

[65] Venter M. (2018). Assessing the Zoonotic Potential of Arboviruses of African Origin. Curr. Opin. Virol..

[66] Kosoy O., Rabe I., Geissler A., Adjemian J., Panella A., Laven J., Basile A.J., Velez J., Griffith K., Wong D. (2016). Serological Survey for Antibodies to Mosquito-Borne Bunyaviruses Among US National Park Service and US Forest Service Employees. Vector-Borne Zoonotic Dis..

[67] Reusken C., van den Wijngaard C., van Beek P., Beer M., Bouwstra R., Godeke G.-J., Isken L., van den Kerkhof H., van Pelt W., van der Poel W. (2012). Lack of Evidence for Zoonotic Transmission of Schmallenberg Virus. Emerg. Infect. Dis..

[68] Boutzoukas A.E., Freedman D.A., Koterba C., Hunt G.W., Mack K., Cass J., Yildiz V.O., de Los Reyes E., Twanow J., Chung M.G. (2023). La Crosse Virus Neuroinvasive Disease in Children: A Contemporary Analysis of Clinical/Neurobehavioral Outcomes and Predictors of Disease Severity. Clin. Infect. Dis..

[69] Da Rosa J.F.T., de Souza W.M., de Pinheiro F.P., Figueiredo M.L., Cardoso J.F., Acrani G.O., Nunes M.R.T. (2017). Oropouche Virus: Clinical, Epidemiological, and Molecular Aspects of a Neglected Orthobunyavirus. Am. J. Trop. Med. Hyg..

[70] Endy T.P., Ryan E.T., Hill D.R., Solomon T., Aronson N.E., Endy T.P. (2020). 36—Viral Febrile Illnesses and Emerging Pathogens. Hunter’s Tropical Medicine and Emerging Infectious Diseases.

[71] Cao-Lormeau V.-M., Musso D. (2014). Emerging Arboviruses in the Pacific. Lancet.

[72] Gould E., Pettersson J., Higgs S., Charrel R., de Lamballerie X. (2017). Emerging Arboviruses: Why Today?. One Health.

